# A Comparasion in Graft Resorption between Three Techniques of Diced Cartilage Using Surgical Blade, Electrical Grinder and Grater in Rabbit

**Published:** 2014-01

**Authors:** Ali Manafi, Mohammad Sabet, Abolhasan Emami, Mohammad Vasei, Jaber Mosavi, Amir Manafi, Zahra Sadat Hamedi, Farzad Manafi, Golnoush Mehrabani, Navid Manafi

**Affiliations:** 1 Department of Plastic Surgery, Tehran University of Medical Sciences, Tehran, Iran;; 2 Department of Pathology, Tehran University of Medical Sciences, Teharn, Iran;; 3 Student Research Committee, Shahid Beheshti University of Medical Sciences, Teharn, Iran;; 4 Student Research Committee, Iran University of Medical Sciences, Teharn, Iran;; 5 Student Research Committee, Shiraz University of Medical Sciences, Shiraz, Iran;; 6 Student Research Committee, Zanjan University of Medical Sciences, Zanjan, Iran

**Keywords:** Graft resorption, Diced cartilage, Blade, Grinder, Grater, Rabbit

## Abstract

**BACKGROUND:**

In recent years, there is an increasing tendency to use diced cartilage grafts in rhinoplasty surgery for improving dorsum contour irregularities. This study was designed to compare graft resorption between three techniques of diced cartilage using surgical blade, electrical grinder and grater in rabbit model.

**METHODS:**

Thirteen New Zealand rabbits were divided into three groups. Three 2×2 cm cartilage specimens were harvested from one of their ears. In group one, the cartilage was diced by use of No:11 surgical blade to o.5 to 1 mm cube pieces. In group two, an electrical grinder was used and in group three, a grater was applied. The grafts were placed in three subcutaneous pockets in the back of rabbits and after 12 weeks, the implants were removed and their weight and volume were recorded and were evaluated by histological techniques.

**RESULTS:**

There was no difference between the three methods in the 3 groups for graft resorption. There was no change in the volume, but the weight showed a decrease in the control group.

**CONCLUSIONS:**

As the histological results had no statistically difference between groups, we may recommend use of these two techniques in reconstructive and in aesthetic cases.

## INTRODUCTION

Autogenous cartilage grafting has been used extensively for augmentation of soft tissue or framework in craniofacial and cosmetic Surgery. Cartilage with its low metabolic rate and avascular structure is a special tissue that its nutrition is provided by tissue fluid diffusion. It has an anaerobic glycolytic activity and O_2_ consumption and it was shown to have a good survival after implantation.^1^

The use of cartilage grafts for reconstruction of craniofacial, nasal and auricular defects in the either form of cartilage grafts or in composite graft form has widespread use. The idea of utilizing cartilage tissue for grafting was previously described.^2^ After introducing the concept of using autogenous cartilage grafting in reconstructive surgery in early 20^th^ century, these grafts had a widespread use because of its availability near the surgical field and good host tolerance.^3^

Crushed cartilage grafts have been used for correction of nasal contour deformities.^3^ Despite improvements in techniques for cartilage grafting, some problems still remained with methods of cartilage wrapping, visibility, graft extrusion and long term integrity of the graft. Graft resorption and unpredictability of results along with donor site limitations were the other potential problems.^4^ In an attempt to address limited availability of cartilage for harvesting, the technique of cartilage dicing into small pieces was introduced^4^ and it was widely used in clinical fields.^5^

The technique had limited use by some plastic surgeons worldwide till 2000. It was shown that using diced cartilage grafts wrapped in oxidized regenerated cellulose (Surgicel) as Turkish delight technique could improve nasal dorsal contour in rhinoplasty.^6^ In this technique, autogenous cartilage that was finely diced into 0.5-1.0 mm cubes, bathed in 1 ml of patients blood and wrapped with surgicel (Ethicon, Somervile, NJ, USA) acted as a re-absorbable oxidized regenerated cellulose product that was frequently used for intra-operative hemostasis. The use of this technique was reported in more than 2000 rhinoplasty cases with long term follow up evaluation.^6^

On the other hand, others reported less success using this approach. This Technique was used in 22 patients and after 3 months, some degree of resorption was seen. Temporalis fascia was used to wrap diced cartilage. In 20 patients, fascia-wrapped diced cartilage was applied but no notable resorption was observed.^7^ Diced cartilage can be used without any wrapping material. The technique is to fill a 1 ml insulin syringe with diced cartilage and direct injection of the cartilage into soft tissue pockets as a filler and augmentation material.^7^

One of the main concerns in cartilage grafting is graft resorption in a long term period. So multiple studies on decreasing of resorption rates have been done including perichondrium preservation,^8^ degree of crushing,^9^ prior history of trauma to the cartilage,^10^ mixing of cartilage with bone dust,^11^ adding fat origin stem cells,^12^^-^^14^ wrapping in fascia,^15^ wrapping in surgicel,^16^ wrapping in Alloderm,^17^ and adding platelet rich plasma (PRP) to diced cartilage.^18^

Utilizing diced cartilage graft technique have some benefits such as being autogenous and elimination of the risk of rejection by host, the graft can be prepared in surgical field, graft harvesting can be done from different donor sites like rib, conchea and septal cartilage, the graft is moldable and can be fitted on the recipient location, and the graft can be reshaped till ten days after operation. Lack of the risk for wrapping of graft, lack of need for use of foreign materials Like K-wire for preserving shape of graft, the resistance of graft to infection (compared with allografts), and the simplicity of correcting graft deformity are some of other benefits of this technique.^15^^-^^18^

Therefore, this study was conducted to compare graft resorption between three techniques of diced cartilage tissue using surgical blade, electrical grinder and grater in rabbit as an animal model.

## MATERIALS AND METHODS

Thirteen New Zealand white rabbits were used as experimental model. The rabbits’ weights ranged from 2000 to 2400 g. The animal study was performed according to guidelines of Iranian Ministry of Health and Medical Education and Helsinki protocol for animal research studies. The Ethics Committee of Tehran University of Medical Sciences approved the study. All of stages of grafts preparation and implantation were performed in two days.

**Fig. 1:**
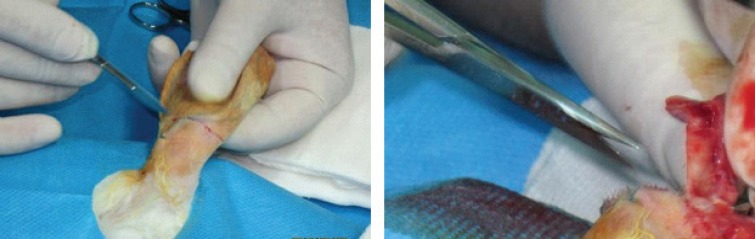
Harvesting cartilage from a rabbit’s ear

Each rabbit underwent general anesthesia with an intra-muscular injection of 15 mg/kg of ketamin hydrochloride and 5 mg/kg of xylazine hydrochloride. As we had two rabbits in each cage, the left ear of one rabbit and the right ear of another rabbit were amputated in each cage (Figure 1).

**Fig. 2:**
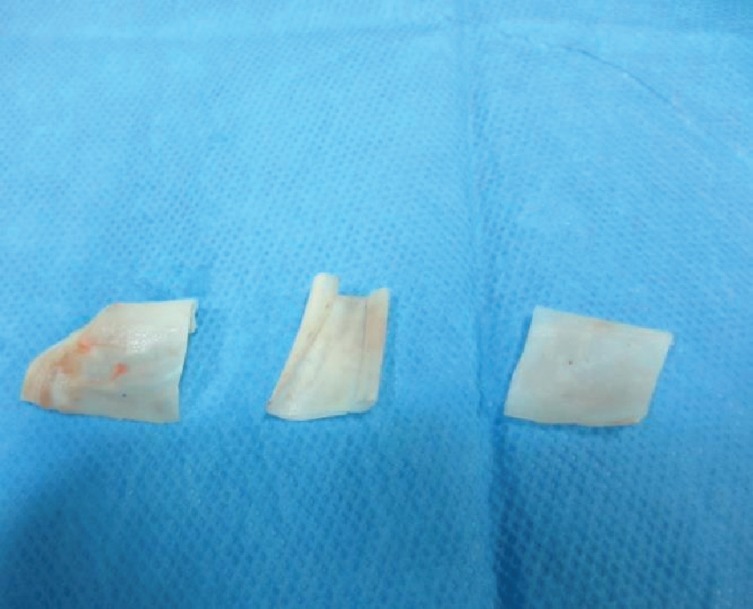
Harvested cartilage divided to three 2×2 cm pieces

**Fig. 3:**
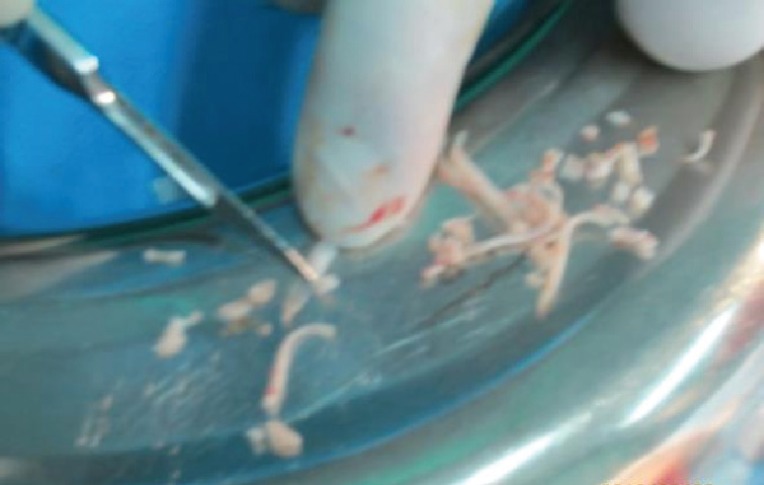
Dicing cartilage with No.11 surgical blade

**Fig. 4:**
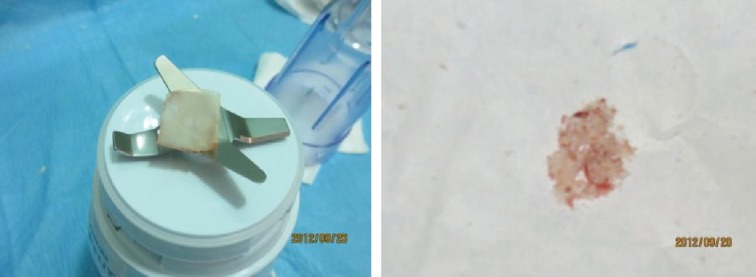
Second piece of cartilage diced with an electrical grinder (Philips, Avente) with 50Hz speed

**Fig. 5:**
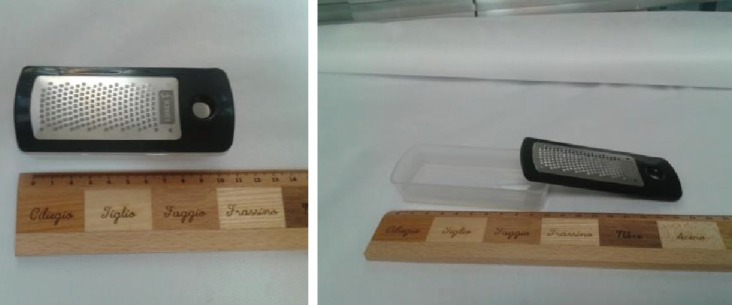
The grater used in study for dicing cartilage with the reservoir beneath it

Cartilage grafts were harvested and dorsal and ventral perichondrial layers were removed from each piece of resected cartilage. They were then harvested and the cartilages were divided into three 2×2 cm pieces (Figure 2). The first piece was diced by use of No.11 surgical blade to 0.5 to 1 mm cube pieces (Group 1, Figure 3). The second piece of cartilage was diced by use of an electrical grinder (Philips. Avente) using a 50 Hz speed (Group 2, Figure 4). The third piece of cartilage was diced into small pieces by use of a grater with a special reservoir beneath it for collecting small pieces (Group 3, Figure 5). Formalin was used for sterility of instruments.

**Fig. 6:**
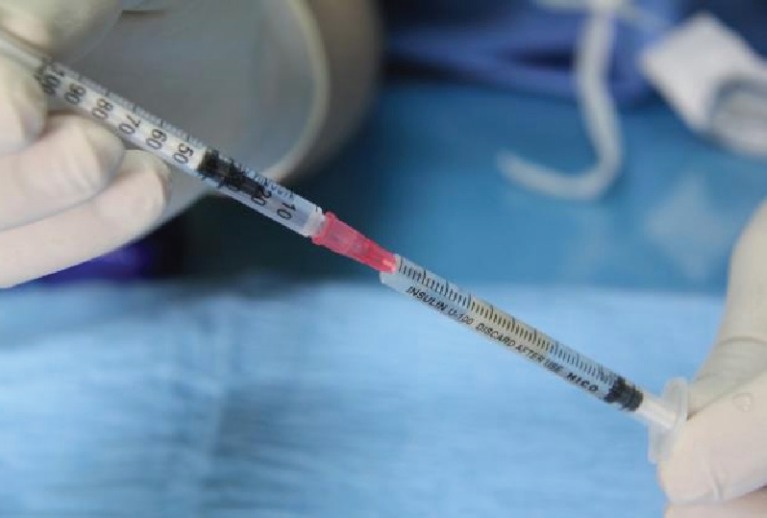
Measurement of volume of specimens

**Fig. 7:**
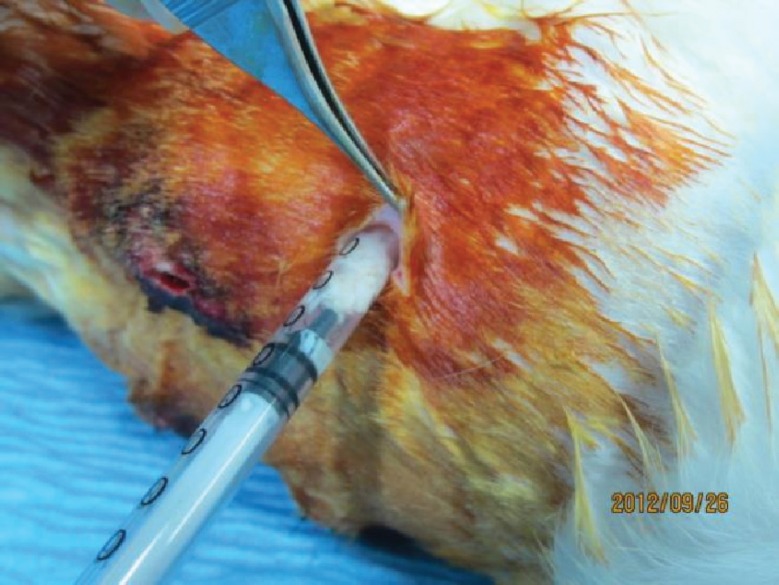
Implantation of grafts in subcutaneous pockets

For volume measurement, specimens were packed into a 1 ml insulin syringe. Another insulin syringe was filled with 1 ml of normal saline solution and then, serum wan added gradually to first syringe until the volume reached to 1 ml level and the remainder of serum in the first syringe was documented as specimen volume (Figure 6). After documentation of data, three subcutaneous pockets were made on the back of each rabbit between panniculus carnosus and deep fascial layers. The cartilage specimens were implanted in the pockets by injection from an insulin syringe. The incision site was closed by nylon 4-0 (Figure 7).

The grafts were divided to three groups. In group 1 as the control group, the cartilage was diced by use of a No.11 surgical blade; in group 2, the cartilage was diced with an electrical grinder and in group 3, the cartilage was diced by use of a grater. After termination of anesthesia, the rabbits were moved to their cages. During postoperative period, 60 mg/kg of ceftriaxon was administrated intramuscularly for 5 days. In 12 weeks period of study, signs of infection were seen in three rabbits and in each group there was one case of wound infection. In the time of graft harvesting, sings of graft resorption was seen in these three grafts. In two of the infected sites, suture line dehiscence was noticed. All rabbits were euthanized at the end of 12 weeks.

**Fig. 8:**
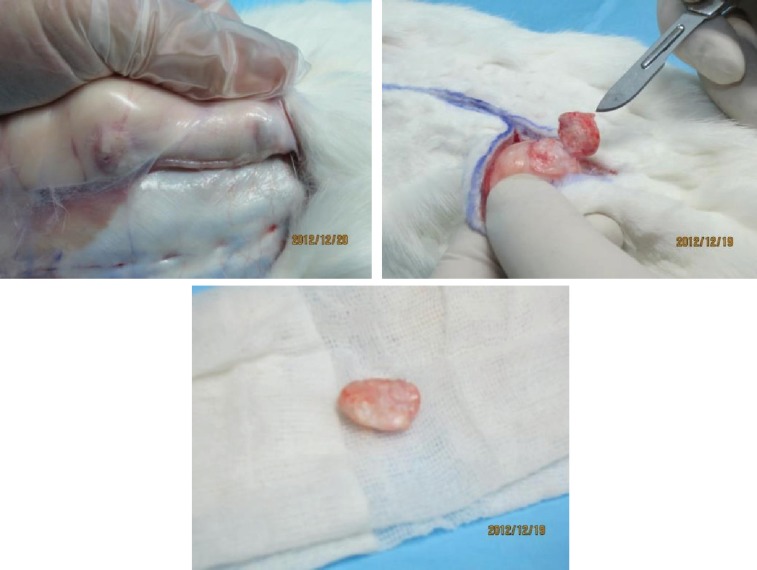
Harvesting the implants after 12 weeks

**Fig. 9:**
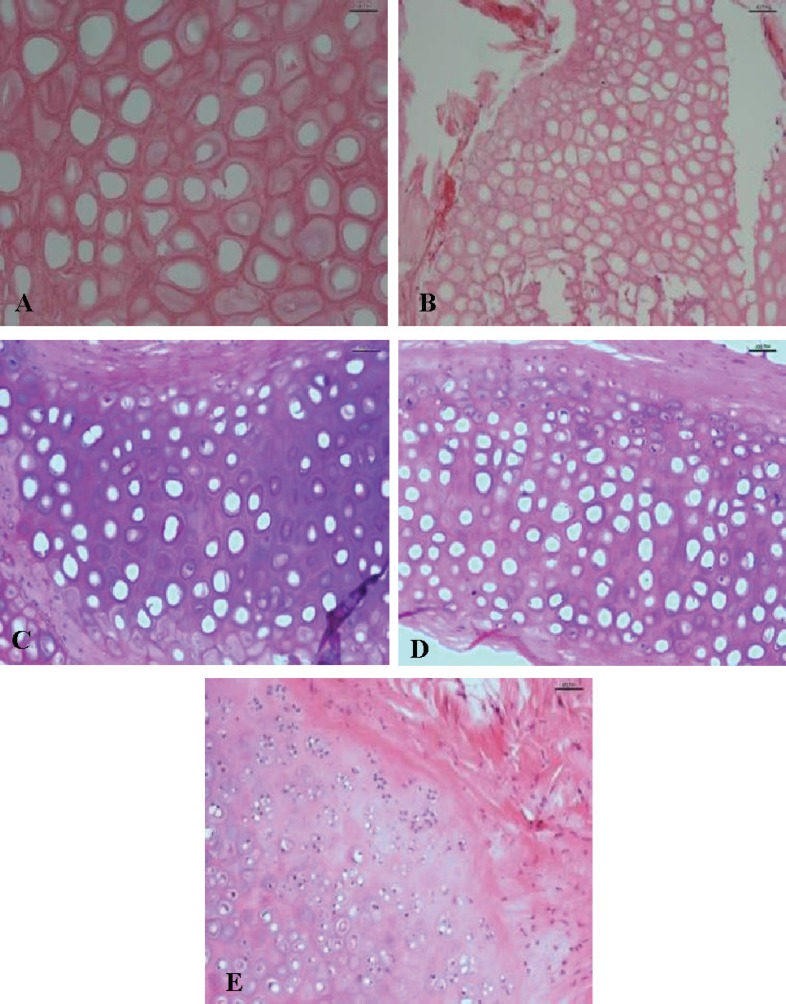
Hematoxylin & eosin staining. A) Total nuclear loss. Score 0 (up-left). B) Near total nuclear loss 10-15% cellularity. Score +1(up-right). C) 40-50% nucleated lacunae: Score +2 (middle-left). D) 60-70% nucleated lacunae: Score +3 (middle-right). E) Above 80% cellularity: Score +4 (low).

The skin was removed at the graft sites and under X4 loop magnification by use of No.15 surgical blade. The grafts were dissected and freed from adjacent surrounding tissues. Diced cartilage specimens were firmly adhered and their separation was not so simple to do (Figure 8). All grafts were weighted and their volumes were measured. The graft specimens were embedded in l0% formalin solution and send for histological evaluation. Three different staining were used for histological evaluation as follows:

***Hematoxilyn and eosin*** ***(H&E):*** The number of nucleated lacunae that took basophilic staining was counted and their percentage in specimen was recorded. Each specimen was scored as 0: nucleated lacunae not seen, +1: 1-25% nucleated lacunae, +2:26-50% nucleated lacunae, +3:51-75% nucleated lacunae, and +4: more than 75% nucleated lacunae seen in specimen (Figure 9).

**Fig. 10:**
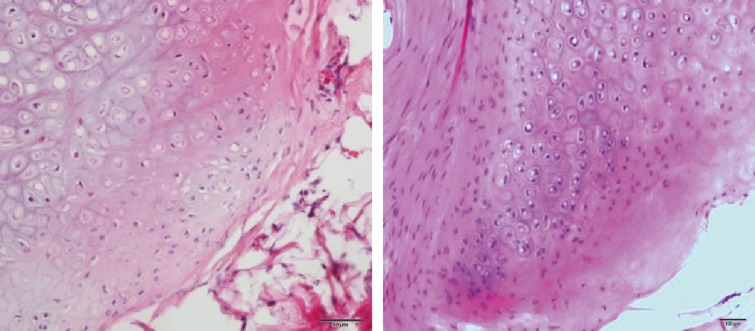
Active proliferation zone

Other histological findings that were evaluated by H&E staining were evidence of active proliferation zones in specimen and this parameter was scored as 0: proliferation zone not seen, +1: rarely seen in one high power field, +2: often seen in each field, and +3: usually seen in every field (Figure 10).

**Fig. 11:**
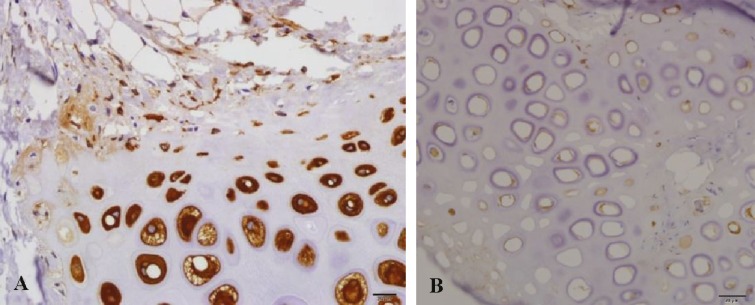
Glial fibrillary acidic protein staining. A) Low staining: score +1 (right). B) Strong staining: Score +2 (left).

***Glial fibrillary acidic protein (GFAP) staining: ***GFAP staining as an immunohistiochemistry staining was used to demonstrate cellular regenerative potential. Brown color staining of specimen was considered as positive. Both peripheral and central zones of graft were evaluated. The scoring was done as 0: negative staining, +1: weak staining, and +2: strong staining (Figure 11).

**Fig. 12:**
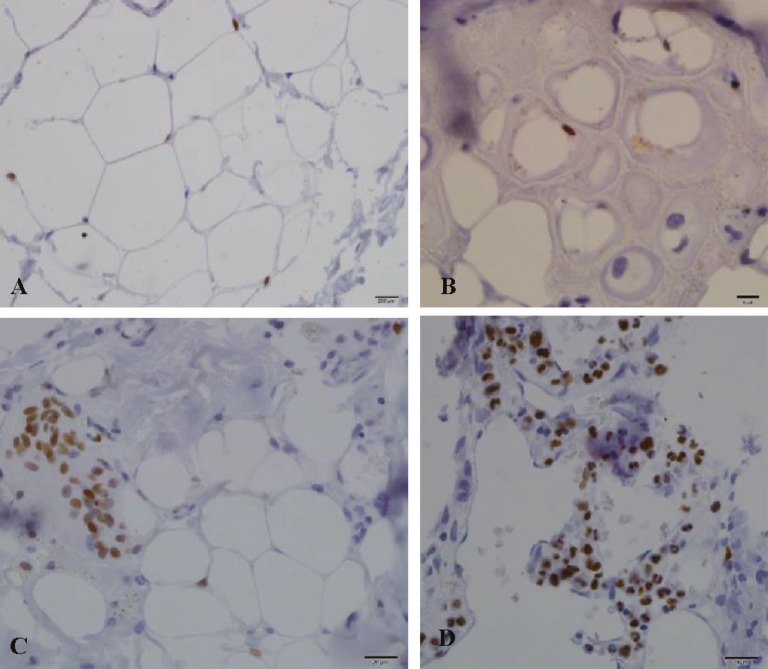
Ki67 staining. A) 1+ score (up-left). B) 2+ score (up-right). C) 3+ score (low-left). D) 4+ score (low-right).

***Ki67 staining: ***This staining was used as an index of mitotic pool in stromal cartilage and demonstrated graft viability and regenerative ability. The graft was scored as 0: negative staining, +1: rarely seen, +2: was seen in every 2 or 3 HPF, +3: less than 50% positive in each HPF, and +4: more than 50% positive in each HPF (Figure 12).

Histological evaluation and staining were done in the university hospital pathological ward (Shariati Hospital) and all of evaluations and scorings were performed by one Pathologist. All analyses were performed using SPSS software (Version 19 for windows statistical package program, Chicago, IL, USA). The values were given as mean±SD. For the comparison between groups mean values, ANOVA test was used. Tukey post HOC test was used to compare three groups’ difference values. P<0.05 was considered significant 

## RESULTS

**Fig. 13:**
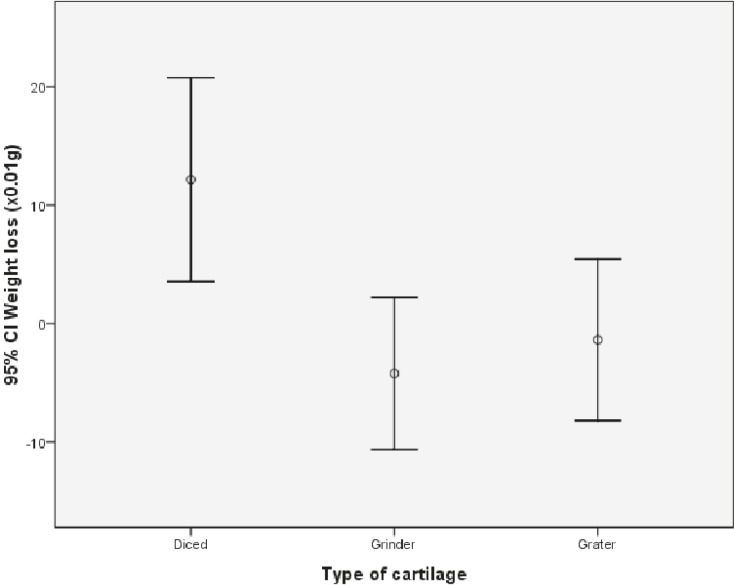
Weight change comparison between groups

In the beginning of study, the average weight of specimens was 0.17 g in group 1, 0.18 g in group 2 and 0.17 g in group 3. After 12 weeks, the mean weight in group 1 reached to 0.29, in group 2 to 0.14 g, and in group 3 to 0.15 g (Table 1). A statistically significant difference was visible between group 1 and the other two groups (P<0.05). Although in group 3, there was less degree of weight change and there was no statistically significant difference between group 2 and 3 regarding weight change (Figure 13).

**Table 1:** Weight and volume means value measurements

	**Diced**	**Grinder**	**Grater**
Initial weight (×0.01 g)	17.0±8.216	18.31±9.13	24.0±8.43
Weight after 3 months (×0.01 g)	29.15±13.91	14.08±7.59	15.15±8.47
Weight loss (×0.01 g)	12.15±14.24	-4.23±10.65	-1.38±11.29
Initial volume (×0.01 cc)	31.15±13.61	22.08±6.58	24.0±8.43
Volume after 12 weeks (×0.01 cc)	34.0±13.42	19.08±6.67	23.77±7.91
Volume loss (×0.01 cc)	2.85±10.43	-3.00±9.19	-23.00±8.59

**Fig. 14:**
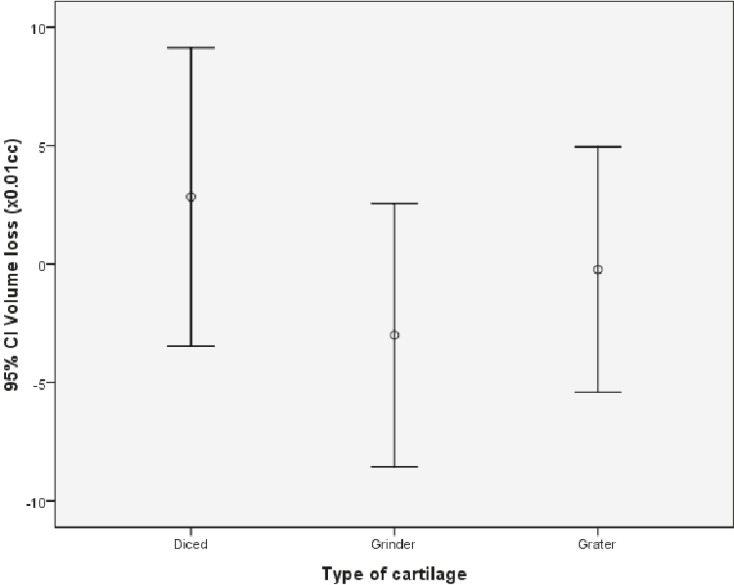
Volume change comparison between groups

Regarding volume, the average initial volume measurement was 0.32 ml in group 1, 0.22 ml in group 2 and 0.24 ml in group 3. After 12 weeks, these measurements were 0.34 ml in group 1, 0.19 ml in group 2, and 0.24 ml in group 3 (Table 1). Among the three groups, no statistical difference was noticed (P>0.05) (Figure 14).

***H&E staining***: The nucleated lacunae showed regeneration and viable cartilage tissue that stained basophilic in H&E sections. The difference between three groups was not statistically significant (P=0.76) (Table 2). Peripheral proliferation zone of chondrocytes did not demonstrate any statistical difference between groups (P=0.40) (Table 2).

**Table 2:** Percent of hematoxylin and eosin chondrecyte nucleation values, hematoxylin & eosin proliferation zone values, glial fibrilary protein (GFAP, peripheral and central zones) staining values, and Ki67 staining value for evaluation and scoring of cartilage

**Indices and scores**	**Diced**	**Grinder**	**Grater**
Hematoxylin and eosin chondrecyte nucleation			
+1	18.2	33.3	30.8
+2	27.3	25	23.1
+3	54.5	41.7	30.8
+4	0	0	15.4
Hematoxyline & eosin proliferation zone			
0	45.5	33.3	61.5
+1	18.2	41.7	23.1
+2	27.3	25	15.4
+3	9.1	0	0
Glial fibrilary protein (peripheral zone)			
0	9.1	53.8	8.3
+1	90.9	46.2	25
+2	0	0	66.7
Glial fibrilary protein (central zone)			
0	36.4	46.2	33.3
+1	36.4	46.2	50
+2	27.3	7.7	16.7
Ki67 staining value			
0	33.3	10	18.2
+2	22.2	50	27.3
+3	22.2	30	45.5
+4	22.2	10	9.1

***GFAP staining: ***Both peripheral and central zone stainings were compared between groups (Table 2). There was not any significant difference between three groups of study. The p value in peripheral zone was 0.14 and in central zone was 0.62.

***Ki67 staining: ***There was no significant statistical difference between groups regarding the mitotic activity of tissue (P=0.17) (Table 2).

## DISCUSSION

Nasal dorsal hump resection sometimes leads to nasal irregularities and converts its smooth contour to an irregular one with sharp edges of bone and cartilage or may also result into an open roof deformity. Especially in thin skinned patients, the edges of underlying skeleton becomes more prominent and eventually gives the patient an “operated–on look” nose. Dorsal nasal grafts are placed under dorsal skin and above osteocartilaginous skeleton to prevent direct contact between skin and underlying irregular dorsum. Some authors have stated the use of autogenous soft tissue grafts such as temporalis fascia and dermal grafts.^17^ Temporalis fascia has low resoption rate but it is tinny and enough augmentation cannot be achieved by use of it.^20^

Alloplastic materials like gelatin film,^21^ silicon,^22^ polyglactin,^23^ Gortex,^24^ and polytetrafluroethylene^25^ have also been advocated for this purpose. However absorbable materials resolve overtime and non-absorbable synthetics were shown to have high rate of exposure and infection.^24^

In contrast to these options, autogenous cartilage is widely accepted as ideal graft material for use in facial reconstructive and cosmetic surgery. However when solid carved pieces of cartilage are used to conceal residual deformities, the edges of on lay graft may cause unsightly irregularities after resolving edema in time. Although cartilage grafts permit shaping to some extent, because of its inherent physical and biomechanical properties, they tend to return to their original condition. This phenomenon is called wrapping which leads to late postoperative deformities.^27^

For breakdown the tendency of cartilage for wrapping, crushing of cartilage was performed. However, still some argument exists about the use of crushed cartilage grafts. It was shown that both crushed and non-crushed cartilage grafts had a success rate from 85.5 to 93.5% when used in rhinoplastic surgeries.^28^ crushed and non-crushed single layer cartilage were used in rabbit model and a volume retention after 90 days for 94.5% of the fresh non-crushed cartilage grafts and 70% of crushed cartilage grafts were noticed. The act of crushing had greatest effect on volume reduction in their study.^29^

Diced cartilage graft is another alternative for smoothing out dorsal irregularities and filling defects. Its use was first introduced in 1943 and has been popularized since 2000. It was shown that diced cartilage grafts were viable and spaces between the small cubes of cartilage were filled by connective tissue.^5^

Diced cartilage is an excellent tool for filling and augmentation purposes, but it may also be difficult to be introduced in the tissue. Different techniques have been presented for this issue. Some investigators implanted diced cartilage into prepared subcutaneous pockets by syringe. Cartilage pieces were wrapped in surgical.^6^ The reports demonstrated the clinical failure of diced cartilage wrapped in surgicel and recommended the use of temporalis fascia for wrapping around cartilage before implantation.^7^ If a large amount of cartilage graft is required, harvesting of rib cartilage is often needed. Diced cartilage grafts can use all excised cartilage pieces with goad viability and less visibility in rhinoplasty.^7^

In this study, we demonstrated that preparing diced cartilage grafts by utilizing an electrical grinder and a grater for dicing ear cartilage of rabbits was possible and cartilage pieces prepared with these techniques had comparable results in term of cellular viability and regeneration potential and the final volume with diced cartilage grafts that was prepared with use of surgical blade.

The comparison of weight between three groups of study revealed a statistically significant difference between group 1 and two other groups. However the amount of weight loss in group 3 was lower but this difference was not statistically significant between groups 2 and 3. On the other hand, there was no difference in volume changes between three groups of study. In other studies like the study in 2005, the degree of volume change had been mentioned as the index of resoption rate in grafts.^9^

In our study we used three different histological staining by utilizing H&E, GAFP and Ki67 stainings. Histological findings in all three groups were comparable and there was no significant statistical difference between three groups for pathologic parameters. The discrepancy between histological findings and weight loss can be explained by probable technical issues in precise weighting of specimens. There were some technical problems in collecting pieces and measuring weight and volume of diced cartilages, according to empty spaces between small pieces (which will be filled by connective tissue) as mentioned before.^30^

No other study was available to compare alternative techniques in dicing cartilage. Most of studies were about comparing viability and resorption of grafts wrapped in different materials like surgicel, fascia and Alloderm.^31^ In 2011, a comparison between grafts was done in 6 different groups, 2×2cm blocks of cartilage were used including one bare cartilage block, one piece wrapped in surgicel, and the third block wrapped in fascia. The other three pieces were diced with surgical blade and used as previous series, in first pocket, and diced cartilage without any extra coverage that was implanted. The second specimen used was wrapped in surgicel and the third group used was wrapped in fascia. All 6 specimens were implanted in subcutaneous pockets in the back of rabbits. After two months, grafts were harvested and results were compared. The least resorption rate and the most viable grafts were in the bare group of grafts without use of any wrapping material. In half of rabbits, autogenous grafts were used and in other half, allograft cartilage was used. It was shown that with removing perichondrium of cartilage, allografts did not create major problems and they had good host tolerance and low antigenicity. In this study, histological evaluation was used for detecting viability and volume change of specimens used for resorption rate.

In 2006, histological analysis was used in diced cartilage grafts for detecting the cause of graft resorption in surgicel wrapped diced cartilage. Different stainings of H&E was used for general condition of cartilage. Trichrome staining was used to demonstrate collagen content of matrix, Van Gieson staining used for detecting elastic fibers in matrix, Safranin-o used as index of proteoglycan content of matrix and glial fibrillary, acidic protein staining for demonstrating regeneration potential of chondrocytes. They showed better results in diced cartilage grafts wrapped in fascia in comparison with surgicel wrapped cartilage.^32^

In 2001, the problem of precise measurement of graft volume was noticed due to tissue in-growth and fibrosis between cartilage pieces. The role of histological evaluation for comparing between diced, crushed and surgicel wrapped diced cartilage grafts were demonstrated. In this study, glial fibrillary acidic protein staining in surgicel wrapped group was negative but bare diced cartilage grafts had positive results.^30^

The results of different degrees of crushing in a seven year follow up of 462 rhinoplasty patients were compared. The cartilage grafts were crushed to four different degrees. They concluded that the degree of crushing applied was important for long term clinical outcome of autogenous crushed cartilage grafts. Slight or moderate crushing of cartilage had good long term results but the resorption rate of significantly crushed grafts was higher (9.1% vs. 13.1%).^33^

In another study, histological parameters were used for evaluation of cartilage grafts. They compared three groups of diced cartilage grafts in rabbits. First group was diced cartilage alone, in the second group, cartilage pieces were wrapped in fascia and in the third group diced cartilage was wrapped in alloderm. They used H&E, Masson trichrome, safranin–o, Van Gieson and GAFP staining. Their results demonstrated that chondrocyte regeneration potential, matrix collagen content and metaplastic bone formation of alloderm treated group showed superior results but the differences were not statistically significant. They concluded that alloderm may be an excellent material for diced cartilage grafting.^17^

Our study about using alternative techniques of diced cartilage preparation with an electrical grinder and a grater demonstrated the possibility and comparability of results of these two methods with conventional technique of dicing cartilage with surgical blade. In spite of reduction in weight of grafts prepared with electrical grinder and grater, the weight reduction was lower in grater group but the difference was not statistically significant. Volume change measurements as the index of graft resorption was in the same range between all three groups.

As the histological results by H&E, GFAP and Ki67 staining had no statistically difference between groups, we may recommend use of these two techniques in reconstructive and in aesthetic cases.

## Footenote

Please cite this paper as:

Manafi A, Sabet M, Emami AH, Vasei M, Mosavi J. A Comparasion in Graft Resorption between Three Techniques of Diced Cartilage Using Surgical Blade, Electrical Grinder and Grater in Rabbit. World J Plast Surg 2014;3(1):52-63.
